# Taurine alleviates oxidative stress in porcine mammary epithelial cells by stimulating the Nrf2‐MAPK signaling pathway

**DOI:** 10.1002/fsn3.3203

**Published:** 2023-01-22

**Authors:** Mengmeng Xu, Long Che, Kaiguo Gao, Li Wang, Xuefen Yang, Xiaolu Wen, Mengyun Li, Zongyong Jiang

**Affiliations:** ^1^ College of Animal Science and Technology Henan University of Animal Husbandry and Economy Zhengzhou China; ^2^ State Key Laboratory of Livestock and poultry Breeding, Key Laboratory of Animal Nutrition and Feed Science in South China, Ministry of Agriculture, Guangdong public Laboratory of Animal Breeding and Nutrition, Guangdong Key Laboratory of Animal Breeding and Nutrition, Institute of Animal Science Guangdong Academy of Agricultural Sciences Guangzhou China

**Keywords:** Nrf2, oxidative stress, porcine mammary epithelial cells, sow

## Abstract

The high incidence of oxidative stress in sows during late gestation and lactation affects mammary gland health, milk yield, and milk quality. Recently, we found that supplementing maternal diets with 1% taurine improved antioxidant capability and enhanced growth performance in offspring; however, the mechanisms underlying these are unknown. This study aimed to investigate the cytoprotective effects and the mechanism of taurine in mitigating oxidative stress in porcine mammary epithelial cells (PMECs). PMECs were pretreated with 0–2.0 mM taurine for 12 h and then subjected to oxidative injury with 500 μM hydrogen peroxide (H_2_O_2_). Pretreatment with taurine attenuated decreased cell viability, enhanced superoxide dismutase, and reduced the intracellular reactive oxygen species accumulation after H_2_O_2_ exposure. Taurine also prevented H_2_O_2_‐induced endoplasmic reticulum stress. Nuclear factor erythroid 2‐related factor 2 (Nrf2) was essential to the cytoprotective effects of taurine on PMECs, as *Nrf2* knockdown significantly inhibited taurine‐induced cytoprotection against oxidative stress. Moreover, we confirmed that *Nrf2* induction by taurine was mediated through the inactivation of the p38/MAPK pathway. Overall, taurine supplementation has beneficial effects on redox balance regulation and may protect against oxidative stress in lactating animals.

## Graphical abstract

Taurine prevented H_2_O_2_‐induced cell apoptosis. Taurine prevented H_2_O_2_‐induced endoplasmic reticulum stress. Taurine supplementation may protect against oxidative stress in lactating animals.

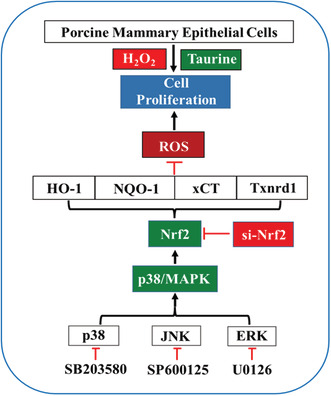

## 1 INTRODUCTION

Mammals can produce and secrete milk, which is vital for the nourishment of preweaning animals (Krogh et al., 2017). During late pregnancy and lactation, the mammary glands are a site of active anabolism and are likely subject to altered redox balance with continuous production of reactive oxygen species (ROS), leading to oxidative stress (Tan et al., 2021). Although a sufficient amount of ROS is believed to be necessary for effective signaling through multiple cellular pathways (D'Autréaux & Toledano, 2007; Sena & Chandel, 2012), excess ROS are generally considered harmful to mammary gland development, milk production, and milk quality (Lee, 2017). Porcine mammary epithelial cells (PMECs) have developed multiple pathway mechanisms to defend against oxidative stress (Xu et al., 2019). Among these, the nuclear factor erythroid 2‐related factor 2 (Nrf2) is one of the best‐characterized transcription factors that regulate the expression of antioxidant proteins to protect against oxidative damage (Sun et al., 2020). Kelch‐like ECH‐associated protein 1 (KEAp1), a cysteine‐rich anchor protein, acts as the main sensor molecule through its effects on Nrf2 within the cytosol (You et al., 2021). Conjugation of excess ROS by cysteine residues results in the suppression of KEAp1‐regulated ubiquitination, thereby leading to Nrf2 nuclear translocation (Nakagami, 2016; Nguyen et al., 2009). After being transported into the nucleus, Nrf2 binds to KEAp1, forming an E3 ubiquitin ligase complex that binds to the antioxidant response element (ARE) for transcriptional activation and the regulation of gene expression for antioxidant proteins. Typical examples of Nrf2‐regulated genes include NADpH‐quinone oxidoreductase 1 (NQO1), heme oxygenase‐1 (HO‐1), thioredoxin reductase (Txnrd), and the cysteine uptake transporter (xCT) (Lee, 2017). Research shows that complex interactions occur between the Nrf2 and MAPK signaling pathways. The MAPK signaling pathway can influence the activity of Nrf2, and Nrf2 can also modulate the activation of MAPK pathway. For example, the functional loss of Nrf2 leads to the activation of MAPK pathway and induces inflammatory responses (Chen et al., 2012). However, forced activation of Nrf2 significantly suppresses Erk1/2 activation (Tan et al., 2011). Therefore, Nrf2 may affect the proliferation or milk secretion of PMECs by regulating the MAPK signaling pathway.

Taurine is a free amino acid found abundantly in animals, which plays a crucial role in a variety of biological processes (Ma et al., 2021). Previous studies show that taurine protects cells against damage caused by the excess production of cytotoxic ROS at sites of inflammation in humans (Kim & Kang, 2015). Additionally, both cell (Wu et al., 2018) and animal studies (Han et al., 2020) have demonstrated the efficient antioxidant properties of taurine. However, its antioxidant role in PMECs is not well understood. Therefore, this study aimed to evaluate the protective effects and possible mechanisms underlying the alleviation of oxidative stress induced by hydrogen peroxide (H_2_O_2_) in PMECs by taurine. Our study may provide scientific guidance for the future application of taurine as an alternative strategy against oxidative stress in lactating animals.

## 2 MATERIALS AND METHODS

### 2.1 Reagents for cell culture

Dulbecco's modified Eagle's Ham/F12 medium (DMEM/F12), trypsin/EDTA, fetal bovine serum (FBS), trypsin/EDTA, antibiotics solution, and sterile phosphate buffer saline were purchased from Invitrogen (Carlsbad, CA). Cell culture dishes were obtained from Corning Inc. (Corning, NY). Taurine, epidermal growth factor (EGF), insulin, hydrocortisone, and other chemicals were supplied by Sigma‐Aldrich (St. Louis, MO). Nrf2 small‐interfering RNA (siRNA) and a negative control (NC) siRNA were designed and synthesized by Genepharma (Shanghai, China). p38 MAPK inhibitor (U0126), JNK inhibitor (Sp600125), and ERK1/2 inhibitor (SB203580) were obtained from Sigma‐Aldrich. The following antibodies were employed in this study: anti‐GRP78, anti‐CHOP, anti‐Nrf2, anti‐HO‐1, anti‐NQO‐1, anti‐Xct, and anti‐Txnrd1 antibodies were purchased from Abcam (Cambridge, UK); anti‐JNK, anti‐P‐JNK anti‐ERK, anti‐P‐ERK, anti‐p38, and anti‐P‐p38 antibodies were purchased from Cell Signaling Technology (Beverly, MA); and anti‐β‐actin antibody was obtained from Amyjet Scientific (Wuhan, China).

### 2.2 Cell culture and treatment

Porcine mammary epithelial cell cultures were prepared as previously described (Che, Xu, Gao, Wang, et al., 2019). Porcine mammary epithelial cells cultures were maintained in DMEM/F12 containing 10% FBS, insulin (5 μg/ml), hydrocortisone (1 μg/ml), 1× PSN antifungal/antibiotics, and 5 ng/ml EGF at 37°C in a humidified atmosphere with 5% CO_2_. The culture medium was replaced every 2 days. Cells were passaged using 0.25% trypsin–EDTA when 90% confluency was achieved, then reseeded (6.0 × 10^3^ cells/well) into 96‐well plates or 2.5 × 10^5^ cells/well were grown in 6‐well plates. To construct an in vitro oxidative stress model, H_2_O_2_ was added to PMECs. H_2_O_2_ (30%) was then diluted to 1 M using 100 μl of 30% H_2_O_2_ and 870.3 μl PBS. The 1 M H_2_O_2_ solution was further diluted with the culture medium at the appropriate concentrations. All H_2_O_2_ solutions were prepared immediately before use. After pretreatment with H_2_O_2_, the cells were treated with the indicated concentrations (0, 0.4, 0.8, 1.2, 1.6, and 2.0 mM) of taurine for 12 h.

### 2.3 Cell viability assay

Cells (6.0 × 10^3^ cells/well) were cultured in a 96‐well culture plate. After incubating for 48 h, CCK‐8 reagent (20 μl/well) was added and then incubated again at 37°C for 2 h, after which the OD_450_ was measured using a microplate reader.

### 2.4 Measurement of intracellular ROS and oxidative stress biomarkers

Reactive oxygen species production was determined using a ROS assay kit (Nanjing Jiancheng Bioengineering Institute, Nanjing, China) or carboxy‐H2DCFDA staining assay. Briefly, following treatment for carboxy‐H2DCFDA staining assay, PMECs were incubated with 200 μl of carboxy‐H2DCFDA at 37°C for 15 min. The cells (1 × 10^6^) were resuspended in PBS and then subjected to flow cytometric analysis. The proportions of fluorescent cells were determined using a FACS Calibur flow cytometer (BD Biosciences, San Diego, CA, USA). T‐SOD activity was evaluated using a commercial kit (Nanjing Jiancheng Bioengineering Institute, Nanjing, China) according to the kit's procedure.

### 2.5 Transient transfection and *Nrf2* siRNA

A total of three candidate siRNAs targeting the coding region of *Nrf2* mRNA and an NC siRNA (Table S1) were synthesized by Genepharma (Shanghai, China). The specificity and effectiveness of the PMEC siRNAs were evaluated by determining Nrf2 protein expression after siRNA transfection for 48 h. The siNrf2‐2 was found to be the most effective and thus selected for further analysis (60% silence, Figure S1). Transfection was conducted with Lipofectamine 3000 reagent RNAiMAX (Invitrogen, Carlsbad, CA, USA) by following the manufacturer's protocols.

### 2.6 MAPK signaling pathway inhibition

Cells (2.5 × 10^5^ cells/well) were cultured in a six‐well culture plate. To determine the effect of MAPK on the Nrf2 signaling pathway, six treatment groups were designed, including a control group (Con), H_2_O_2_, taurine + H_2_O_2_, taurine + H_2_O_2_ + U0126, taurine + H_2_O_2_ + SP600125, and taurine + H_2_O_2_ + SB203580. After the PMECs were cultured for 48 h, protein analysis was carried out using western blotting.

### 2.7 2RNA isolation and mRNA expression analysis

RNA was isolated by Trizol reagent (Invitrogen, Carlsbad, CA, USA) and an RNeasy Mini kit (RR037A; Takara Bio, Kusatsu, Japan) in accordance with the manufacturer's instructions. cDNA synthesis was performed with reagents from TaKaRa Biotechnology (Kusatsu, Japan). The real‐time PCR was conducted on 7900HT Fast Real‐Time PCR System (Thermo Fisher Scientific) using SYBR Green Real‐Time PCR reagent (RR820A; Takara Bio). After normalization to β‐actin, the relative expression level of each gene was conducted using the cycle threshold (2^−△△Ct^) method. The primer sequences used are shown in Table S2.

### 2.8 Western blotting

Cell lysates were harvested with cell scrapers and then centrifuged at 12,000 × *g* for 10 min at 4°C. Total protein was extracted with ice‐cold RIPA buffer (Beyotime), and the protein concentrations were determined using a BCA assay kit (Thermo Scientific, Waltham, MA). After mixing with Laemmli sample buffer, the samples were heated at 100°C for 10 min. The protein samples were then separated through 6% or 10% SDS‐PAGE at 110 V for 60 min or 70 V for 40 min before being transferred onto a PVDF membrane at 250 mA for 150 min or 90 min. After blocking with QuickBlockTM Western (Beyotime) at room temperature for 1 h, the membrane was incubated overnight at 4°C with the primary antibodies. After rinsing three times with Tris‐buffered saline with Triton X‐100 for 10 min, the membrane was incubated with the corresponding HRP‐conjugated secondary antibodies at room temperature for 1 h. The membrane was then washed three times for 10 min each, and the immunoreactivity was visualized with chemiluminescent HRP substrate (Millipore, Billerica, MA) using a VersaDoc imaging system (Bio‐Rad, Hercules, CA). The band intensities were obtained with ImageJ software after normalization to β‐actin.

### 2.9 Statistical analyses

All values are shown as mean ± SEM. Statistical tests were conducted with SPSS statistics software (v. 19.0 for windows, SPSS; IBM SPSS Company, Chicago, IL, USA). The significant differences in the assay values of the cell cultures were evaluated using the Student's *t*‐test or one‐way ANOVA followed by the Student–Newman–Keuls test. *p* < .05 was deemed statistical significance.

## 3 RESULTS

### 3.1 Establishment of an oxidative stress model

**FIGURE 1:**
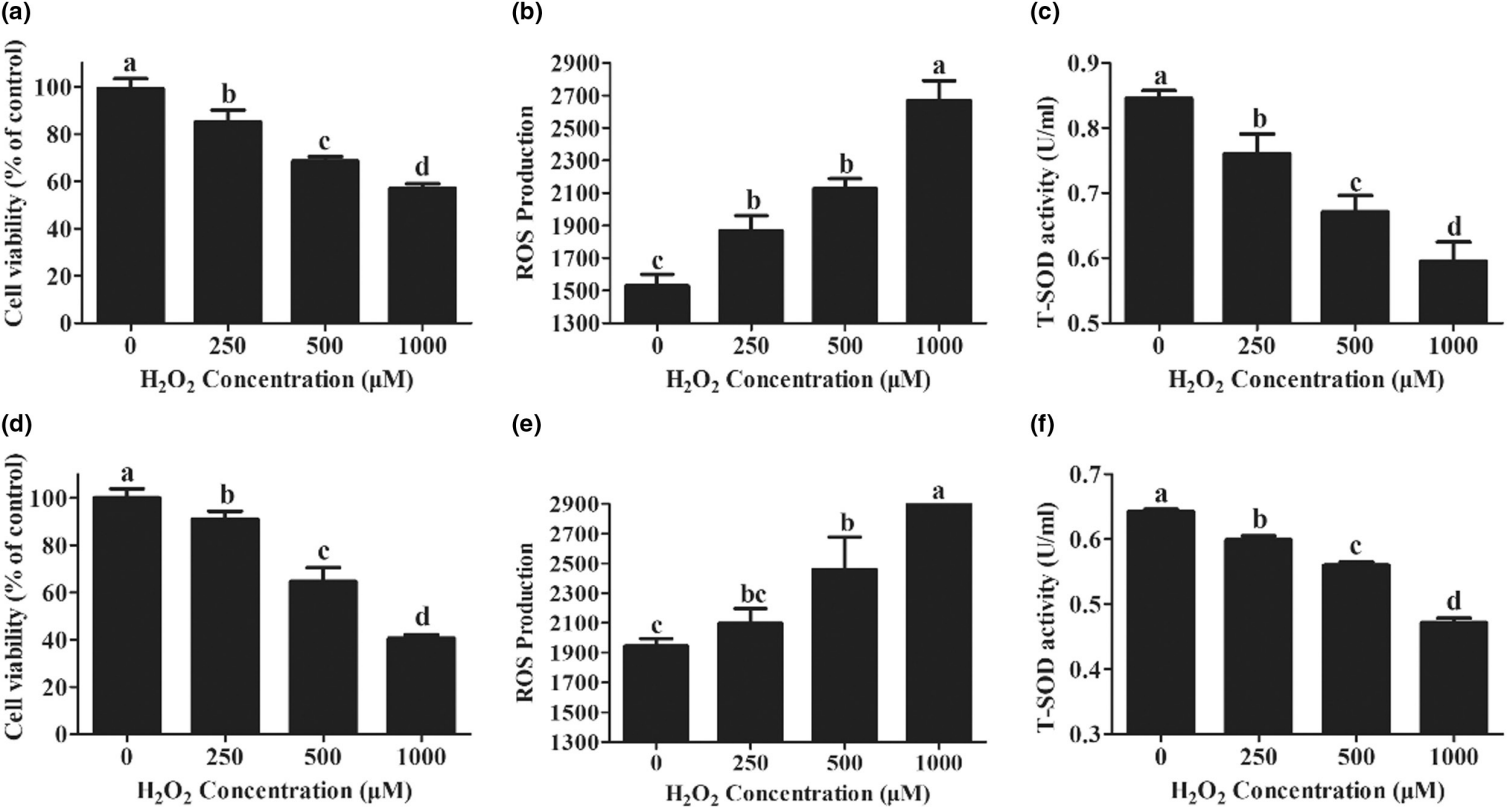
Effects of H_2_O_2_ on the viability, ROS production, and T‐SOD activity of PMECs: (a) Cell viability at 12 h after H_2_O_2_ treatment; (b) ROS concentration at 12 h after H_2_O_2_ treatment; (c) T‐SOD activity at 12 h after H_2_O_2_ treatment; (d) Cell viability 24 h after H_2_O_2_ treatment; (e) ROS concentration at 24 h after H_2_O_2_ treatment; (f) T‐SOD activity 24 h after H_2_O_2_ treatment. Data are shown as mean ± SEM. Means not sharing the same letter are different (*p* < .05).

To determine suitable concentrations of H_2_O_2_ for subsequent experiments, we treated PMECs with different concentrations of H_2_O_2_ and measured the oxidative stress index of the treated cells. Exposure of PMECs to 0–1000 μM H_2_O_2_ for 12 h or 24 h reduced cell viability (Figure 1a, d) while sharply increasing ROS production in a concentration‐dependent fashion (Figure 1b, e). Treatment with 500 μM H_2_O_2_ for 12 h resulted in an approximate 40% reduction in live cells, accompanied by decreased and increased levels of T‐SOD (Figure 1f) and ROS (Figure 1e), respectively, compared to the control. Based on these results, we used 500 μM H_2_O_2_ for 12 h to induce oxidative stress.

### 3.2 Taurine attenuates H_2_O_2_ ‐induced oxidative stress

**FIGURE 2:**
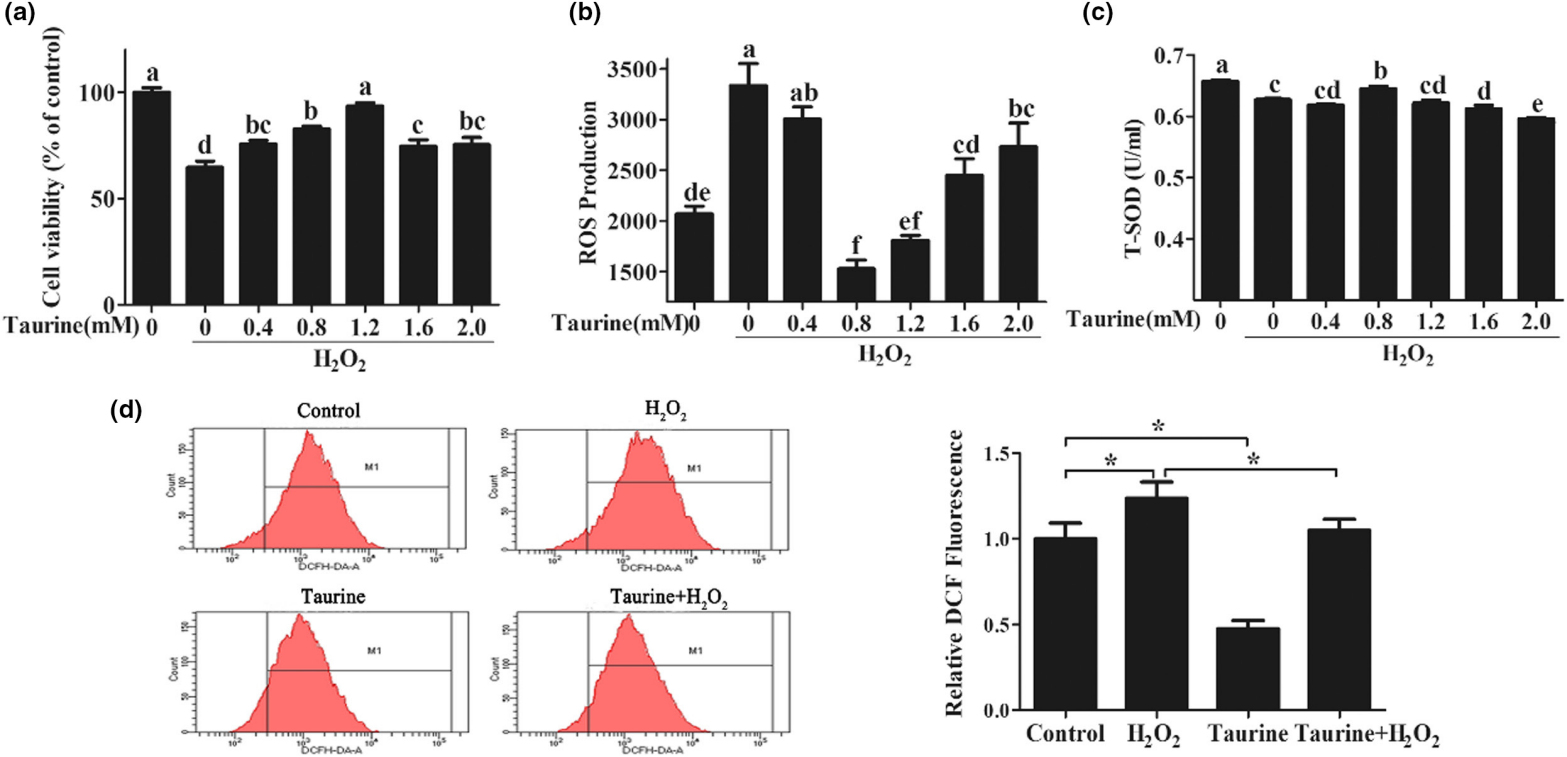
Effects of taurine against H_2_O_2_‐induced oxidative stress: (a) Cell viability; (b) ROS concentration determined using an ROS assay kit; (c) T‐SOD activity; (d) ROS concentration determined using carboxy‐H2DCF‐DA‐staining assay. Data are shown as mean ± SEM. Means not sharing the same letter are different (*p* < .05); *Means significantly different from untreated cells (*p* < .05).

To verify the hypothesis that taurine can alleviate oxidative stress in PMECs, taurine (0–2 mM) was added to the culture 12 h prior to adding 500 μM H_2_O_2_. The results show that exposure of PMECs to 0–1.2 mM taurine for 24 h prior to H_2_O_2_ treatment reduced cell death in a concentration‐dependent fashion (Figure 2a). In addition, 0.8 to 1.2 mM taurine significantly decreased H_2_O_2_‐induced ROS levels in PMECs (*p* < .05; Figure 2b), and the least response was observed with 1.2 mM taurine. Moreover, 0.8 mM taurine significantly increased T‐SOD activity in PMECs compared with H_2_O_2_ treatment (*p* < .05; Figure 2c). We then assessed the protective effect of 1.2 mM taurine against H_2_O_2_‐triggered injury in PMECs. The data showed that 1.2 mM taurine significantly decreased H_2_O_2_‐induced ROS levels in PMECs (Figure 2d).

### 3.3 Taurine improved antioxidant system

**FIGURE 3:**
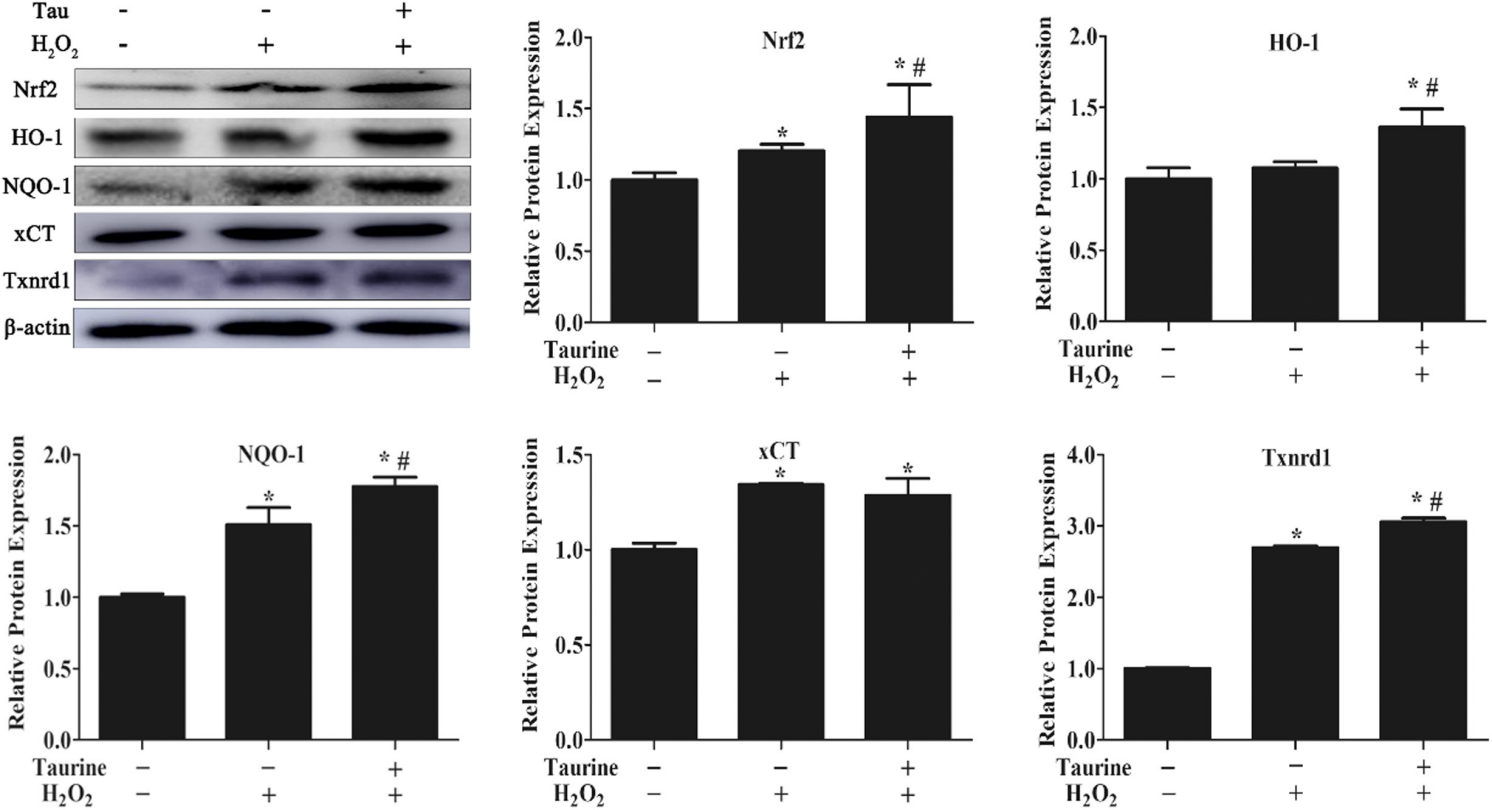
Effects of taurine on protein expression in the Nrf2 antioxidant system under normal and oxidative conditions. Data are shown as mean ± SEM. *Means significantly different from untreated cells. #Means significantly different from H_2_O_2_‐treated cells (*p* < .05).

PMECs were incubated for different periods of time (24 h) with or without 1.2 mM taurine and then incubated for 12 h with or without 500 μM H_2_O_2_. Upon H_2_O_2_ exposure, the cells had a significant increase in protein expressions of the Nrf2 antioxidant system, including that of Nrf2, xCT, NQO‐1, HO‐1, and Txnrd1, compared to Con (*p* < .05; Figure 3). In addition, the NQO‐1, HO‐1, and xCT protein levels were significantly elevated upon treatment with taurine prior to H_2_O_2_ treatment compared to H_2_O_2_ treatment only (*p* < .05; Figure 3).

### 3.4 Taurine alleviates H_2_O_2_ ‐induced endoplasmic reticulum stress in PMECs

**FIGURE 4:**
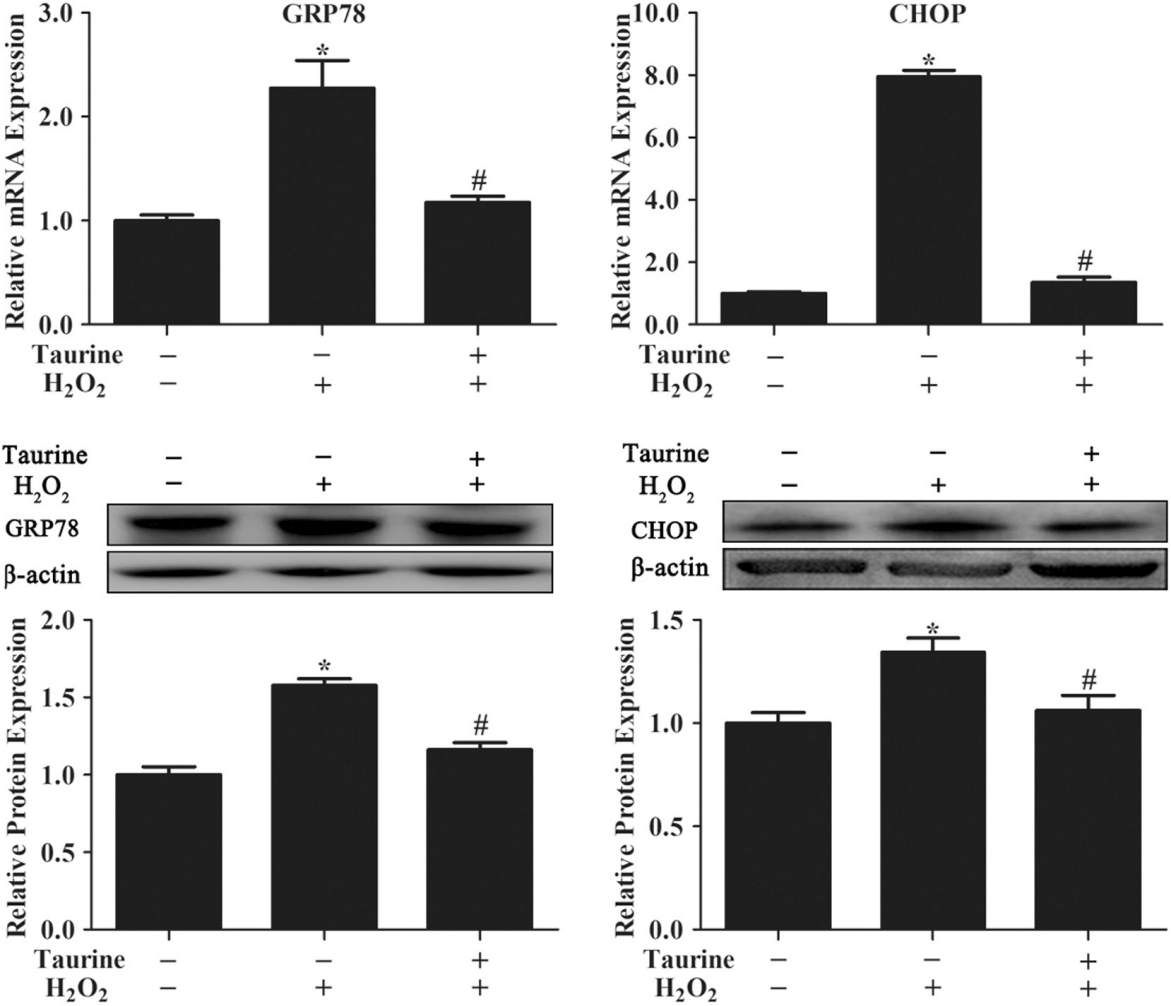
Taurine‐induced gene and protein expression of endoplasmic reticulum stress and apoptosis under normal and oxidative conditions. Data are shown as mean ± SEM. *Means significantly different from untreated cells. #Means significantly different from H_2_O_2_‐treated cells (*p* < .05).

In PMECs, H_2_O_2_ strongly enhanced the mRNA expression and protein levels of C/EBp homologous protein and glucose‐regulated protein 78 (GRP78) (Figure 4). After pretreatment with 1.2 mM taurine, the H_2_O_2_‐triggered mRNA levels of *CHOP* and *GRP78* were remarkably decreased (*p* < .05; Figure 4a, b). Western blotting analysis showed significant increases in GRP78 and CHOP protein levels within H_2_O_2_‐treated PMECs (*p* < .05; Figure 4c, d). However, after pretreatment with 1.2 mM taurine, the H_2_O_2_‐triggered protein levels of CHOP and GRP 78 were markedly reduced (*p* < .05; Figure 4c, d).

### 3.5 The antioxidative stress effects of taurine are dependent on Nrf2 overexpression in PMECs

**FIGURE 5:**
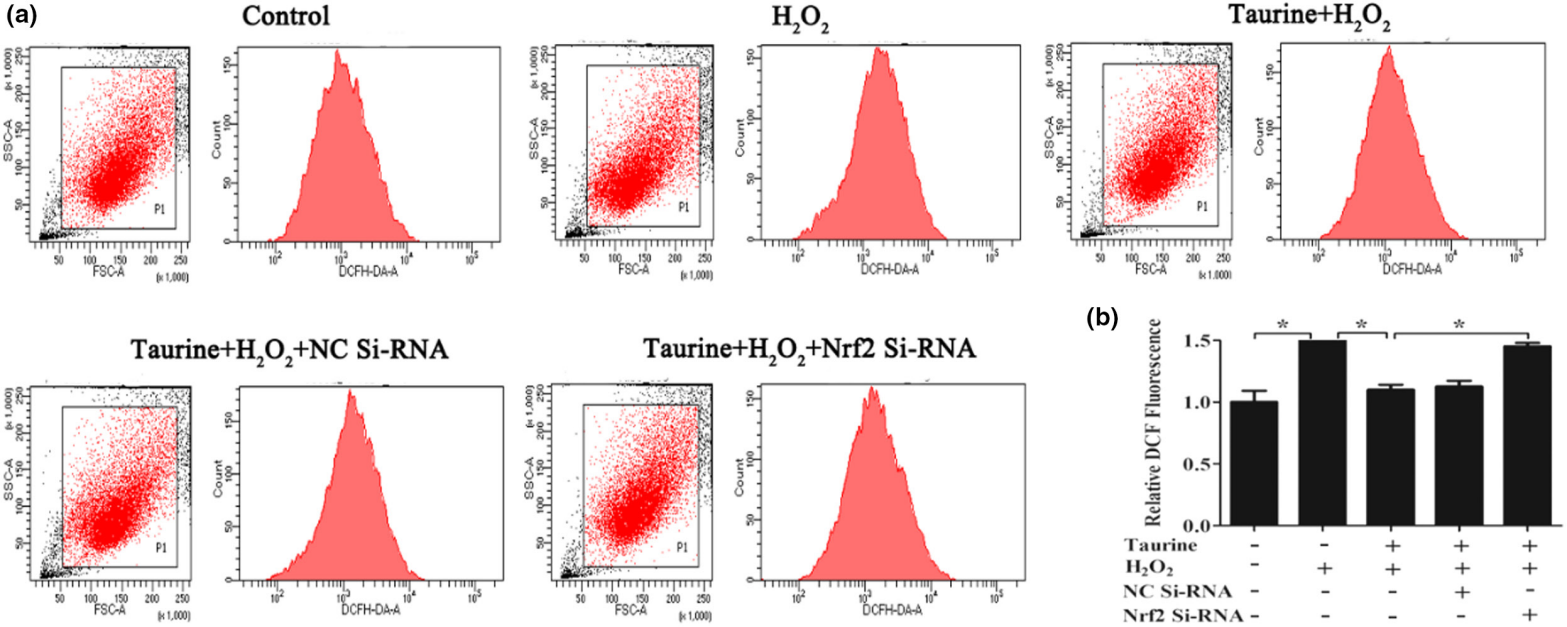
Effects of taurine against H_2_O_2_‐induced ROS production. ROS concentration was determined using a carboxy‐H2DCF‐DA‐staining assay. Data are shown as mean ± SEM. *Means significantly different (*p* < .05).

**FIGURE 6:**
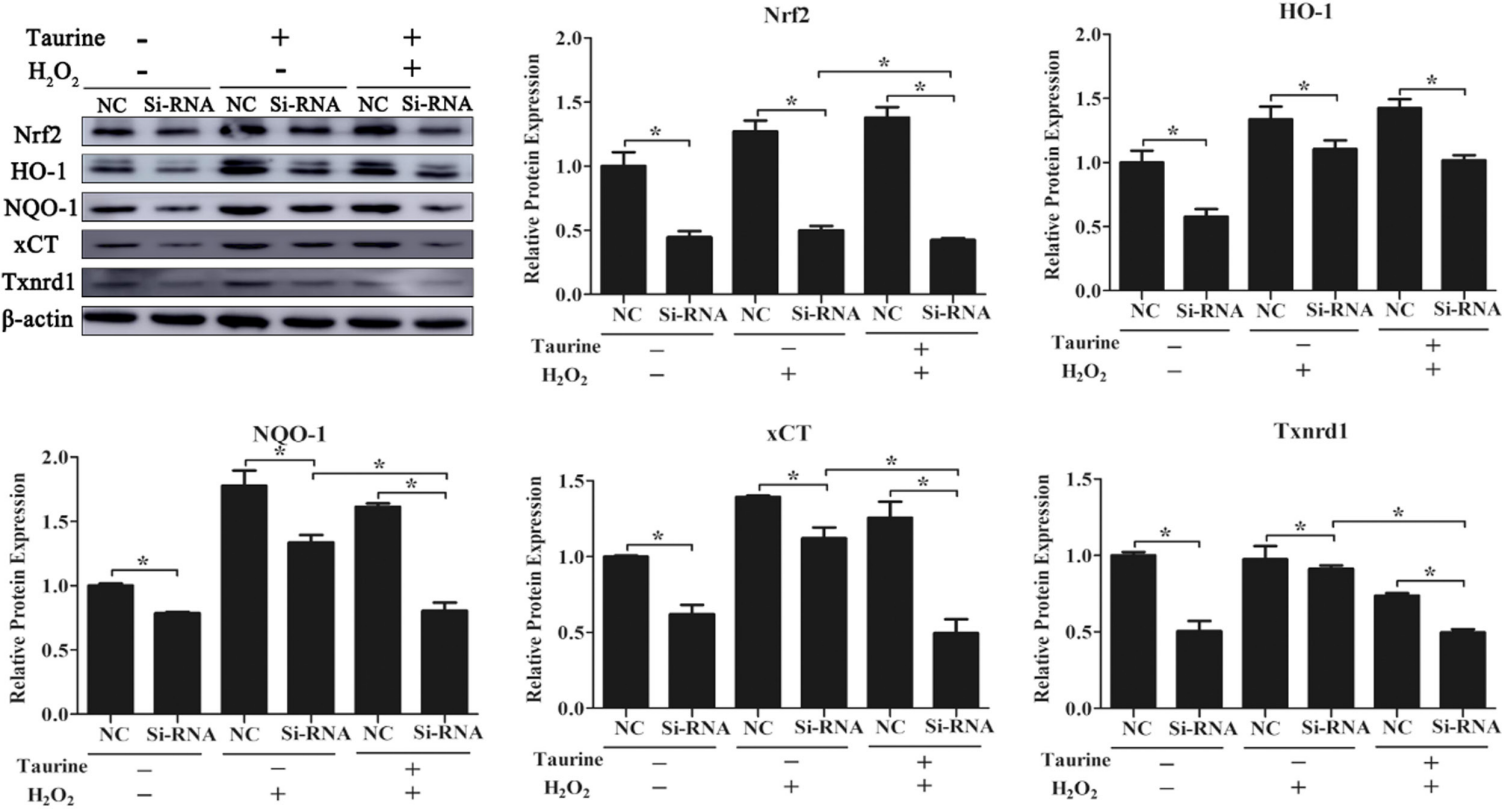
The cytoprotective effects of taurine against oxidative stress are dependent on Nrf2 induction in PMEC. Data are shown as mean ± SEM. *Means significantly different (*p* < .05).

**FIGURE 7:**
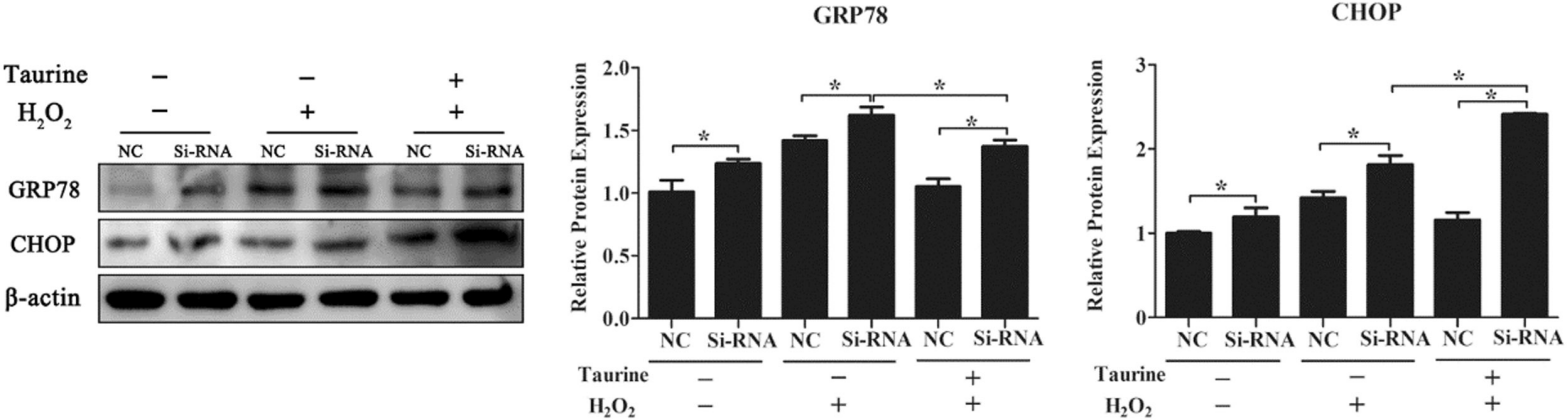
The effect of taurine in reducing endoplasmic reticulum stress and apoptosis is dependent on Nrf2 induction in PMECs. Data are shown as mean ± SEM. *Means significantly different from untreated cells (*p* < .05).

To explore whether Nrf2 can regulate the antioxidative stress effects of taurine, PMECs were transfected with *Nrf2* siRNA. After 48 h of transfection, an obvious decrease in the Nrf2 protein level was found in PMECs in all groups compared to the group transfected with an NC siRNA (*p* < .05; Figure 5b). After Nrf2 knockdown, there was an obvious increase in the ROS level in the PMECs exposed to H_2_O_2_ or taurine (*p* < .05; Figure 5a). Additionally, the levels of Nrf2, HO‐1, NQO‐1, xCT, and Txnrd proteins were significantly decreased in *Nrf2* siRNA‐transfected cells compared to the NC siRNA‐transfected cells (*p* < .05; Figure 6). Furthermore, it was observed that *Nrf2* knockdown could dramatically increase the expression levels of CHOP and GRP78 in H_2_O_2_‐challenged cells (*p* < .05; Figure 7).

### 3.6 Effect of MAPK pathway on PMECs after H_2_O_2_ exposure and taurine treatment

**FIGURE 8:**
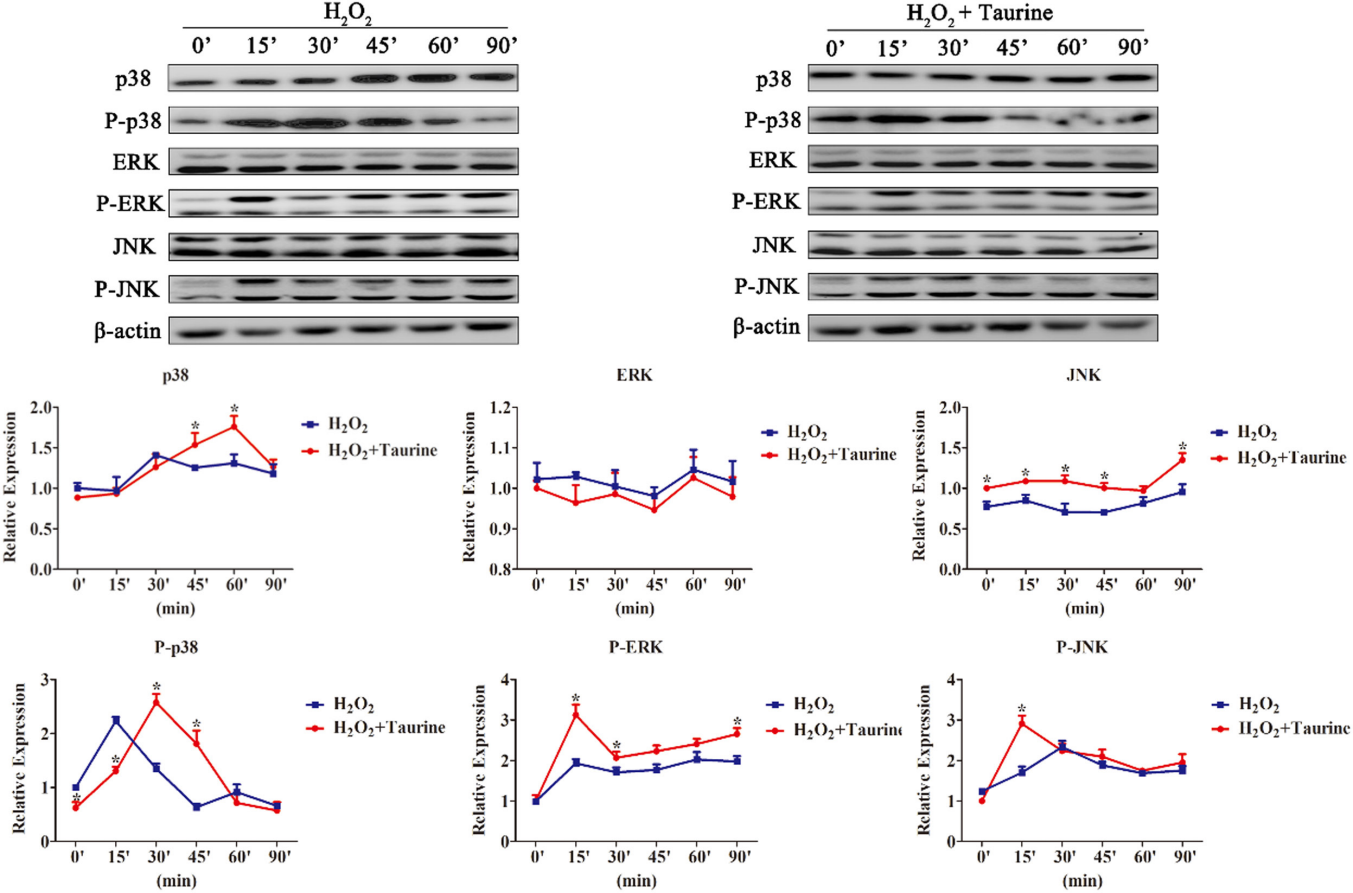
Taurine‐modulated H_2_O_2_ induced phosphorylation of the MAPK pathway in a time‐dependent manner. Data are shown as mean ± SEM. *Means significantly different from untreated cells (*p* < .05).

**FIGURE 9:**
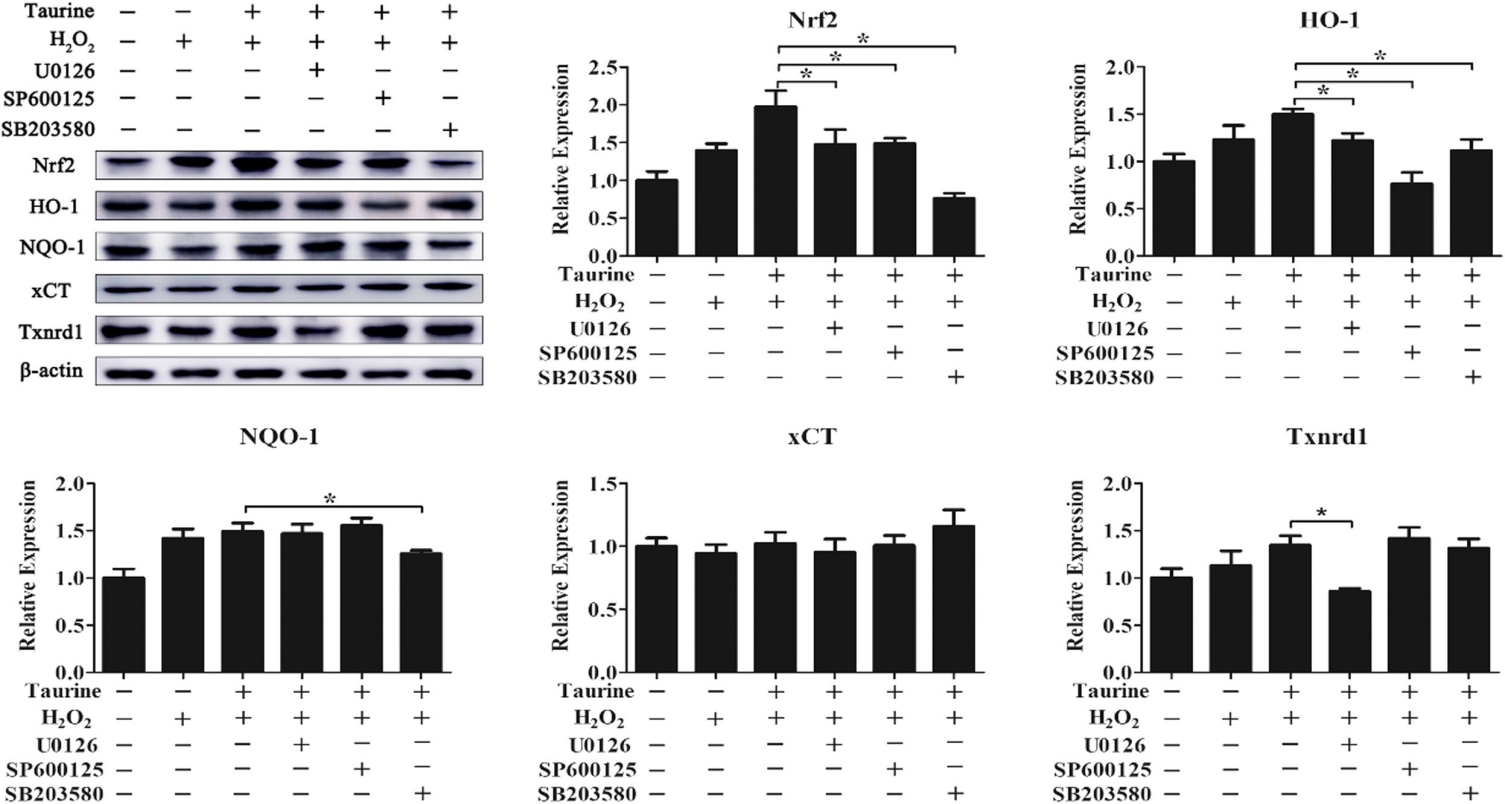
Inhibition of MAPK affects the activation of Nrf2 signaling by taurine. Data are shown as mean ± SEM. *Means significantly different from untreated cells (*p* < .05).

As MAPK regulates Nrf2 and cellular antioxidant responses in many cell types, we hypothesized that taurine would decrease Nrf2‐mediated upregulation in response to oxidative stress by activating the MAPK pathway. Western blotting analysis showed that p38, ERK1/2, and JNK1/2 were rapidly phosphorylated after H_2_O_2_ treatment. More importantly, pretreatment with 1.2 mM taurine resulted in increased phosphorylation of p38, JNK1/2, and ERK1/2 (*p* < .05; Figure 8). We used the following selective inhibitors for MAPK signaling pathways in PMEC cultures: U126 (ERK1/2 inhibitor), Sp600125 (JNK inhibitor), and SB203580 (p38 MAPK inhibitor). Taurine‐induced Nrf2 protein expression was potently inhibited by all MAPK signaling pathway inhibitors (*p* < .05; Figure 9).

## 4 DISCUSSION

Cells accumulate free radical species, such as alkyl and hydroxyl radicals, and lead to oxidative, which can be prevented by antioxidants. Antioxidant defense responses are important for protecting living organisms against toxicants (Plotnikov et al., 2019). In gilts, the mammary glands undergo rapid metabolic changes to allow abundant secretion, with the onset of the lactogenic process beginning around day 90 of gestation (Ji et al., 2006). After parturition, the mammary gland still remains in an anabolic state as the synthesis of milk is required for the offspring (Berchieri‐Ronchi et al., 2011). Evidently, the anabolic state contributes to the generation of free radical species, and the induction of oxidative stress results in the mammary gland, sustaining an oxidative state over time (Shen et al., 2015). The loss of total antioxidant capacity is associated with reduced health status and immune response in gilts (Li et al., 2021). Therefore, dietary supplementation with exogenous antioxidants during the lactation period may protect the mammary glands from oxidative stress.

In the previous study, we observed that taurine exhibits a protective function against oxidative stress by decreasing ROS concentrations and increasing antioxidant enzymes (Xu et al., 2019). Our results are consistent with the previous report that taurine can induce autophagy and inhibit oxidative stress in mice Leydig cells (Yahyavy et al., 2020). To elucidate the mechanisms of taurine‐dependent regulation of oxidative stress, PMEC, a commonly used cell model to evaluate milk synthesis (Che, Xu, Gao, Zhu, et al., 2019; Ma et al., 2018) and antioxidant response, was applied in this study. Our results have provided evidence that taurine directly exerts potent antioxidative potential in an Nrf2‐dependent manner.

To study the function of taurine under oxidative stress conditions, an in vitro oxidative stress model of PMEC was constructed by challenging H_2_O_2_. It was observed that incubating PMEC with 500 μM H_2_O_2_ for 24 h markedly altered cell morphology, increased ROS production, reduced cell viability, and promoted cell apoptosis. These findings are in line with several studies on bovine mammary epithelial cells (Rebucci et al., 2013; Sun et al., 2021). Similarly, numerous studies have documented that persistent ROS generation and oxidative stress can lead to cell injury and eventually cell apoptosis (Pasciu et al., 2010). The protective effects of taurine on H_2_O_2_‐triggered cytotoxicity may be related to the suppression of ROS accumulation and cell apoptosis in PMECs.

Growing evidence has suggested that the cytoprotective effects of taurine are associated with its ability to enhance endoplasmic reticulum function (Chian et al., 2012). The endoplasmic reticulum plays crucial roles in cell apoptosis, free radical production, and energy metabolism (Yang et al., 2022). Endoplasmic reticulum dysfunction could lead to a disruption of electron flow in the electron transport chain, resulting in excessive production of mitochondrial ROS (Murphy, 2013). The endoplasmic reticulum dysfunction in the mammary gland may be associated with stress and apoptosis via the regulation of GRP78 responsive to stress. Elevated levels of GRP78 have been confirmed in pathological conditions and serves as prosurvival factors to mediate cell death by activating CHOP (Ayaub et al., 2016; Zheng et al., 2014). Our results showed that expressions of CHOP and GRP78 were strongly upregulated by endoplasmic reticulum stress induced by H_2_O_2_ treatment, which may result in cell death. However, the pretreatment with taurine restores the oxidative damage caused by H_2_O_2_ treatment. Taurine has differential roles in various cell lines in terms of cell apoptosis (Wu et al., 2019; Xing et al., 2021). Numerous studies have linked oxidative stress to protein misfolding in the endoplasmic reticulum (Subhankar et al., 2011; Tyo et al., 2012), and it has also been shown that the damaged mitochondria might excessively produce reactive oxygen species causing impairment in endoplasmic reticulum protein folding (Yuzefovych et al., 2013), which finally contributed to enhanced apoptosis.

As a transcription factor, Nrf2 plays an essential role in regulating the expression of antioxidant enzymes via interacting with ARE and protecting cells against oxidative stress (Ma, 2013). NQO‐1 and HO‐1 are two major phase II enzymes that promote antioxidant defense (Zhai et al., 2013). The cystine transporter, xCT, and cystine/cysteine cycling can regulate cell defense and alter the redox state of the cell (Meira et al., 2021). Knockout of *Nrf2* in mice can lead to an increase in oxidative stress by downregulating the expression of ARE‐related antioxidant genes (Ma, 2013). In this work, we determined whether taurine can protect PMEC against oxidative stress via Nrf2 signaling. It was observed that taurine treatment enhanced the protein expressions of Nrf2 in PMEC as expected. Then, we assessed the expressions of antioxidant/detoxificant proteins responsible for antioxidative stress and apoptosis in PMEC. We found that taurine affected the protein expression of the downstream antioxidant proteins of the Nrf2 pathway including GPx, GR, SOD1, NQO‐1, xCT, SOD2, Txnrd1, and HO‐1. The upregulated protein levels of SOD2, NQO‐1, HO‐1, NQO‐1, xCT, and Txnrd1 in PMEC by taurine under normal and oxidative stress conditions were also confirmed in our study. These results demonstrated that taurine may restore the cell's redox state by upregulating these antioxidant genes and proteins in PMEC. In addition, the knockdown of *Nrf2* attenuated the upregulation of xCT, Txnrd‐1, and HO‐1 by taurine under H_2_O_2_ exposure or normal conditions, indicating that Nrf2 could mediate the upregulated expression of these genes by taurine. *Nrf2* knockdown in PMEC could elevate the expressions of CHOP, GPR78, and Bax under oxidative stress, suggesting a potential association among Nrf2 activation, endoplasmic reticulum stress, and cell apoptosis. This is consistent with the previous study (Colovic et al., 2017) that taurine may elevate antioxidant defense activities by attenuating the loss of antioxidant enzymes and increasing antioxidant proteins.

Oxidative stress can affect cell proliferation by activating the p38‐MAPK pathway (Palin et al., 2019). Both ERK and p38‐MAPK pathways can be activated by Nrf2. Chen et al. (2012) demonstrated that curcumin could induce the activation of Nrf2 in a p38‐dependent fashion (Chen et al., 2012). Ho et al. (2012) also found that diallyl sulfide could activate Nrf2‐driven antioxidant enzymes through the p38 pathway (Ho et al., 2012). Similarly, our findings suggest taurine prevents oxidative stress induced by H_2_O_2_ via Nrf2 activation, which may be dependent on the downregulation of p38‐MAPK pathway in PMEC.

## 5 CONCLUSIONS

In summary, our study provides the first in vitro evidence on the roles of taurine in protection against oxidative stress in lactating gilts. Taurine treatment protected PMECs against H_2_O_2_‐triggered oxidative stress by activating Nrf2 and scavenging ROS in a MAPK‐dependent fashion. Additionally, the beneficial effect of taurine on PMECs involves the reduction in endoplasmic reticulum stress and cell apoptosis in H_2_O_2_‐challenged PMECs.

## CONFLICT OF INTEREST

All authors read and approved the final manuscript. The authors declare that there are no conflicts of interest.

## ETHICAL APPROVAL

All experimental procedures followed the current law regarding animal protection (Ethic Approval Code: 5YXK2016‐0165) and were approved by the Guide for the Care and Use of Laboratory Animals prepared by the Animal Care and Use Committee of Guangdong Academy of Agricultural Sciences.

## CONSENT FOR PUBLICATION

The authors approved the consent for publication.

## Supporting information


Figure S1

Tables S1‐S2

## Data Availability

The data used to support the findings of this study are available from the corresponding author upon request.
