# Supplementary material for: Taurine alleviates oxidative stress in porcine mammary epithelial cells by stimulating the Nrf2‐MAPK signaling pathway
Source: Food Sci Nutr. 2023 Jan 22;11(4):1736–46. doi: 10.1002/fsn3.3203 (PMC10084955; doi:10.1002/fsn3.3203)
Supplement: Supplementary file 1 — Figure S1 [file FSN3-11-1736-s002.docx]

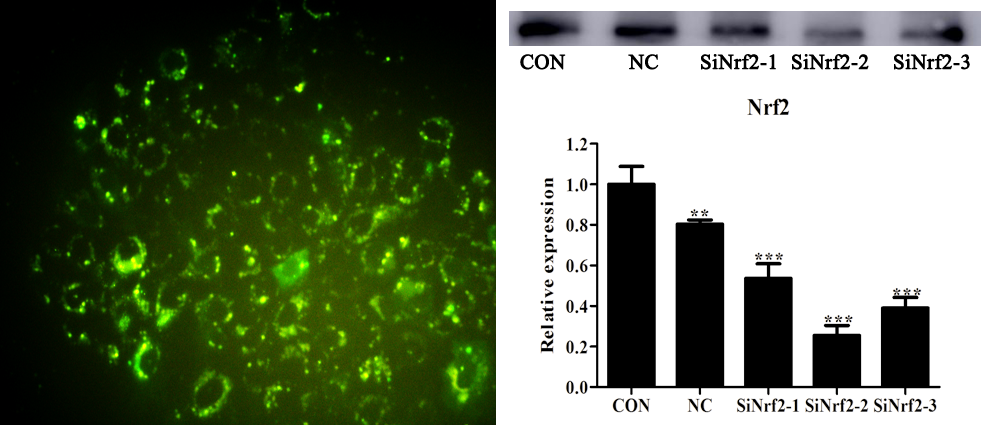


**Supplementary Fig.1.** Gene knockdown efficiency of candidate siRNAs in PMECs. PMECs were transfected with 50 nM negative control siRNA-FAM (NC-FAM), negative control siRNA (NC) and Nrf2 candidate siRNAs for 48h, respectively. Cell transfection efficiency was confirmed by green fluoresce, from the cells transfected with NC-FAM (left panel). The knockdown efficiency of candidate Nrf2 siRNAs in PMEC was confirmed by Nrf2 protein expression analysis after 48 h trasfection (right panel).The protein expressions were measured by western blots, as described in Section 2. Results are shown as mean ± SEM from three independent experiments and each value expressed as a target gene expression ratios comparing with the NC group. * Significantly different from CON control cells.
