# Supplementary material for: Taurine alleviates oxidative stress in porcine mammary epithelial cells by stimulating the Nrf2‐MAPK signaling pathway
Source: Food Sci Nutr. 2023 Jan 22;11(4):1736–46. doi: 10.1002/fsn3.3203 (PMC10084955; doi:10.1002/fsn3.3203)
Supplement: Supplementary file 2 — Tables S1‐S2 [file FSN3-11-1736-s001.docx]

**Supplementary Table 1** Sequences of siRNA duplex used for RNAi.

| siRNA ID | Target position | Sequence* | | | |
| --- | --- | --- | --- | --- | --- |
| siNrf2-1 | 620-642 | S 5': |  | GCCCAUUGAUCUCUCUGAU | dTdT |
|  |  | AS 3': | dTdT | AUCAGAGAGAUCAAUGGGC |  |
| siNrf2-2 | 492-514 | S 5': |  | CCAGAACACUCAGUGGAAU | dTdT |
|  |  | AS 3': | dTdT | AUUCCACUGAGUGUUCUGG |  |
| siNrf2-3 | 1434-1456 | S 5': |  | GCCUAUAAGUCCCGGUCAU | dTdT |
|  |  | AS 3': | dTdT | AUGACCGGGACUUAUAGGC |  |
| NC/NC-FAM | / | S 5': |  | UUCUCCGAACGUGUCACGU | dTdT |
|  |  | AS 3': | dTdT | AAGAGGCUUGCACAGUGCA |  |

* S=sense, AS=anti-sense

**Supplementary Table 2** Primer sequences of the target and reference genes.

| Gene | Primers | Sequence | Accession number | Product size (bp) |
| --- | --- | --- | --- | --- |
| GRP78 | Forward | 5’-AACCAAGGACGCTGGAACTATT-3’ | X92446.1 | 181 |
|  | Reverse | 5’-AACACCAGGATGTTCTTCTCCC-3’ |  |  |
| CHOP | Forward | 5’-GCTGTCTCCACCCATGTTAGAA-3’ | FJ587986.1 | 92 |
|  | Reverse | 5’-CATCCATGTAACTATGCAGCGC-3’ |  |  |
|  | Reverse | 5’-ATCCTCTGCAGCTCCATGTTAC-3’ |  |  |
| β-actin | Forward  Reverse | 5’-GGCCGCACCACTGGCATTGTCAT-3’  5’-AGGTCCAGACGCAGGATGGCG-3’ | DQ845171.1 | 104 |
